# Interventions and Programs Using Native Foods to Promote Health: A Scoping Review

**DOI:** 10.3390/nu16234222

**Published:** 2024-12-06

**Authors:** Carla Vanessa Alves Lopes, Julianna Lys de Sousa Alves Neri, John Hunter, Rimante Ronto, Seema Mihrshahi

**Affiliations:** 1 Department of Health Sciences, Faculty of Medicine, Health and Human Sciences, Macquarie University, Macquarie Park, NSW 2109, Australia; 2 Department of Indigenous and Health Sciences, School of Medical, University of Wollongong, Northfields Ave, Wollongong, NSW 2522, Australia

**Keywords:** native food, traditional food, health promotion, Indigenous peoples, First Nations peoples

## Abstract

(1) Background: Native foods are essential for promoting health, sustainability, and the resilience of Indigenous communities. They contribute to biodiversity, are adapted to local ecosystems, and support cultural identity. This study aims to identify and describe strategies and health outcomes from programs and interventions using native foods to promote health or address food insecurity. (2) Methods: A scoping review was conducted using five databases, including studies that implemented interventions using native foods exclusively or combined with introduced foods (non-native). The review included studies involving human participants and reporting outcomes related to health, nutritional improvements, food security, or cultural impacts. (3) Results: Nineteen studies were included. Most interventions (*n* = 16) combined native foods with introduced healthy foods and implemented multicomponent strategies to promote their intake (*n* = 15), such as educational sessions, food distribution, gardening, cooking activities, community events, media campaigns, and policy changes. Of the 19 studies, 12 explicitly used a participatory method. Reported outcomes included improved dietary intake and nutrient intake, such as increased intake of vitamin A, calcium, and iron; enhanced knowledge about native foods and healthy eating; improved health; positive cultural impacts; and enhanced food security. (4) Conclusions: The results from this scoping review suggest that interventions using native foods show promising results in improving health, nutritional outcomes, cultural identity, and food security, highlighting their potential for broader public health applications and the value of participatory approaches for sustainable interventions.

## 1. Introduction

Native foods, recognised for their nutrient-rich properties, are vital for supporting biodiversity, sustainability, and the resilience of Indigenous communities [1]. Environmentally, native foods are adapted to local ecosystems, using less water and agricultural inputs such as fertilisers and pesticides, which helps mitigate climate change and promotes ecological balance [2]. From a health perspective, native foods are typically rich in essential nutrients, antioxidants, and phytochemicals, which are linked to preventing chronic diseases such as diabetes and cardiovascular diseases [1,2]. Additionally, these foods are minimally processed, which contributes to their nutrient-rich properties [2]. Indigenous communities apply traditional methods such as grinding seeds and cooking and leaching some nuts only for detoxification purposes and make foods more digestible and palatable [2].

Despite this recognition, there are still challenges to preserving and using native foods and the Indigenous food knowledge associated with them [3]. This has contributed to a rise in diet-related diseases and a significant loss of food diversity in Indigenous communities [4]. Indigenous peoples, who constitute only 5% of the global population, account for 15% of global poverty [5]. These communities represent a diversity of languages, cultures, and histories [6], yet share common challenges with socioeconomic status and health, experiencing significantly poorer health outcomes compared to non-Indigenous groups [7,8,9,10,11]. Additionally, systemic marginalisation and discrimination contribute to these health inequities [12]. 

A systematic review and meta-analysis by Shahunja et al. [7] found that Indigenous populations from countries such as Australia, the USA, Canada, New Zealand, and India have twice the likelihood of experiencing multimorbidity compared to non-Indigenous populations. Another systematic review focused on Canadian Indigenous peoples revealed a disproportionate impact of diabetes, with prevalence rates almost twice as high as in the general population [8]. In some remote Northern Territory communities in Australia, the prevalence of diabetes in adults was 28.6% in 2018–2019 [10]. Carman et al. [9] analysed a national survey of Australian individuals, finding that Aboriginal respondents had a two-fold higher prevalence of mental–physical multimorbidity (16.1% vs. 8.1%). Food insecurity also disproportionately affects Aboriginal and Torres Strait Islander peoples compared to non-Indigenous Australians (22% vs. 3.7%) [13]. In Canada, 52% of Inuit ages 25 and older experienced food insecurity in 2012 [14], and in 2017–2018, food insecurity rates were two to six times higher among Indigenous households than other Canadian households [15]. 

These health disparities are rooted in historical and systemic factors such as colonisation, displacement from traditional lands, and policies that disrupt Indigenous food systems [16]. The Australian Institute of Health and Welfare identifies numerous determinants impacting health disparities, including socioeconomic, environmental, health risk, and historical factors [11]. For example, employment rates are lower among First Nations peoples compared to non-Indigenous Australians (56% vs. 78%), which may directly affecting food security [11]. Colonisation is a historical factor that has deeply impacted the culture and health of First Nations communities [11]. Violence, epidemic diseases, and land occupation have caused a loss of cultural connections to land, food, family, and community, resulting in ongoing intergenerational trauma linked to poor physical and mental health outcomes [11,17,18,19]. In Australia, First Nations peoples were forcibly removed from their traditional lands and native foods when colonisers imposed Western diets consisting of flour, sugar, tea, and salted meats [20]. Suppression of native food consumption was part of broader assimilation policies that undermined food sovereignty, disrupted the transmission of traditional food knowledge, and led to a decline in health and wellbeing in these communities [20].

To address the impacts of social inequities, racism, and colonisation trauma, promoting cultural connectedness is essential for improving health outcomes [8,21]. Engaging community members in decision making, traditional practices, and contact with land through culturally safe approaches can strengthen cultural identity, connectedness, and community resilience [8,21]. Revitalising Indigenous food practices by incorporating native foods into community diets is a pathway to enhancing sustainability, food security, and health outcomes in these communities [1,2,4,22]. Egeland and Harrison [23] reviewed various programs and interventions integrating native foods and traditional food practices in Indigenous communities. They found that community-led initiatives and policies centred around native foods can support Indigenous sovereignty and self-determination, thereby addressing health disparities and improving food security [23].

Food sovereignty is the right of Indigenous peoples to define and control their own food systems and promote health through culturally relevant practices [24,25,26], ensuring democratic ownership of food resources [27]. This right is supported by the United Nations Declaration on the Rights of Indigenous Peoples (UNDRIP), which, since 2007, has recognised the importance of revitalising cultural practices and native foods and affirmed Indigenous peoples’ right to make decisions in all policies or research impacting their lives [28]. Since then, many research projects and policies involving Indigenous communities have employed participatory methods. Participatory approaches, such as Community-Based Participatory Research (CBPR), co-design, and co-production, involve researchers, policymakers, and community members working collaboratively and sharing power and decisions throughout the project—from design to implementation, evaluation, and dissemination of results [29,30]. This scoping review aims to identify and describe strategies and health outcomes from programs and interventions implemented using native foods to promote health or to address food insecurity. 

The terms “traditional food,” “native food,” “Indigenous food,” and “local food” are frequently used in the context of health and sustainable food systems. While they share similarities, each has distinct meanings that reflect historical, cultural, and geographical contexts. Native food refers to plants and animals Indigenous to a specific region and adapted to local environments [31,32]. The term is sometimes used interchangeably with Indigenous food, but the latter encompasses broader cultural practices, including spiritual beliefs and ecological knowledge [33]. Traditional food refers to foods consumed over generations with specific preparation methods and cultural significance [34]. Local food emphasises geographic proximity and promotes consuming foods produced close to where they are grown to reduce the carbon footprint associated with transportation [35].

In this review, we predominantly use the term *native food*, as the interventions examined are based on Indigenous communities and involve foods native to their locations. However, considering the interchangeable nature of these concepts in the literature, we retained the terms used by each study in the summary table, with many using “traditional foods.” Similarly, we use the terms *Indigenous* and *First Nations peoples* to describe pre-colonial Indigenous countries with independent languages and identities. Within an Australian context, the term “Aboriginal” refers to all Indigenous peoples of mainland Australia and Tasmania, while “Torres Strait Islander” identifies those groups indigenous to Australia from the islands of the Torres Strait.

## 2. Materials and Methods

### 2.1. Study Design

This scoping review followed the Prisma Extension for Scoping Reviews (PRISMA-ScR) [36] and the five-stage framework developed by Arksey and O’Malley, which involves (1) defining the research question; (2) finding relevant studies; (3) selecting the studies according to the inclusion criteria; (4) organising and categorising the data by key themes; and (5) summarising and presenting the results [37]. The review was registered in PROSPERO (CRD42023463624).

### 2.2. Search Strategy 

A search strategy was developed (Appendix A Table A1) to identify studies that assessed interventions using native foods to promote health. Five electronic databases were accessed to search for relevant studies, which included Scopus, Embase, Web of Science Core Collection, Medline, and PubMed. Key words were applied based on the two main concepts: native food and intervention. The search strategy used for all databases is shown in Table A1. 

### 2.3. Eligibility—PICOS Criteria

The search was limited to results published in English and Portuguese, as we wanted to expand the scope of our review and capture a broader range of studies, considering that our two reviewers are proficient in these languages. No exclusion criteria were applied for the publication date. All peer-reviewed experimental studies on humans were included. Reviews and conference abstracts were excluded. 

The PICOS criteria for inclusion and exclusion of studies were applied as follows: (1) Participants (P): studies were included if they presented the intervention on humans and excluded if the intervention was conducted on animals, such as lab interventions. No age or other specific criteria were applied. (2) Interventions (I): studies used any native food, exclusively or combined with introduced foods, and in different ways, such as nutrition education, cooking, or gardening, that aimed to promote or maintain health or address food insecurity. (3) Comparators (C): no intervention (control group) and the use of introduced foods were considered for inclusion. (4) Outcomes: the primary outcomes included the evaluation of actual or intended consumption, purchase or gardening of native foods, improvements in health and nutritional status, and impacts on food security. The secondary outcomes included the knowledge enhancement of native foods and their health benefits and perceptions of the intervention, including cultural impacts, providing qualitative insights into its effectiveness and acceptance. 

### 2.4. Study Selection

**Figure 1:**
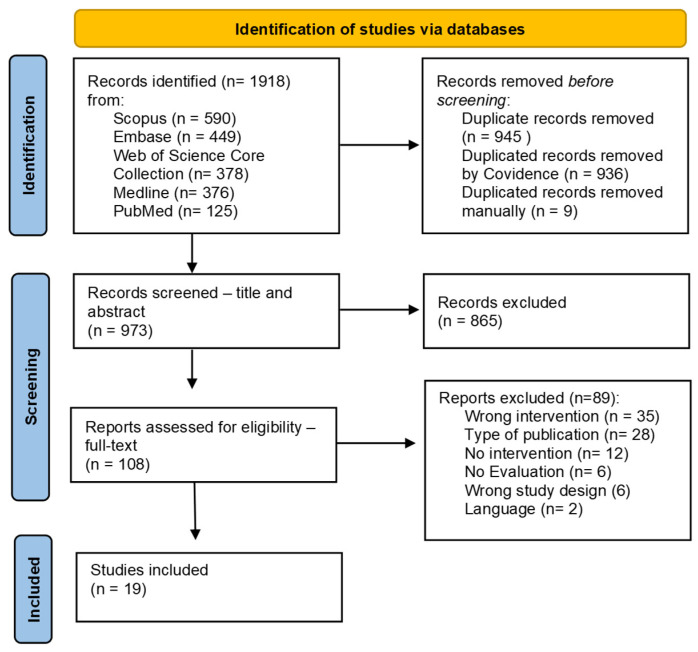
Flowchart of identification and screening process for selection of records assessing interventions and programs using native foods to promote health.

The Covidence platform was used for the study management process. Two authors (CL and JL) independently screened the references’ titles and abstracts. At the second stage, the authors evaluated the full texts according to the eligibility criteria. Any discrepancies between the two reviewers were resolved through consensus or a third reviewer. The flowchart in Figure 1 details the identification and screening process for including the papers. 

### 2.5. Data Extraction and Synthesis

Two researchers (CL and JL) independently extracted relevant data from the included papers using a template developed by the research team on Covidence, which included title, author(s), year of publication, country, aim of the study, study design, methods, intervention details, native food used, population description, inclusion and exclusion criteria, recruitment details, sample size, outcomes, and results. Disagreements were resolved by consensus or by consultation with a third researcher (SM). There was a substantial heterogeneity among the interventions and reported outcomes of the included studies, which made it challenging to conduct a quantitative synthesis or meta-analysis. This heterogeneity is particularly expected in the context of native foods, as there are limited studies available on this topic. Therefore, we opted to use a narrative synthesis to explore and describe the diversity of interventions and outcomes, offering an important first overview of the type of interventions and outcomes related to native foods. 

To analyse whether the studies included a participatory approach, two researchers (CL and JL) independently extracted relevant data for explicit mention of Community-Based Participatory Research (CBPR), Community Advisory Boards (CABs), Community Advisory Groups (CAGs), co-design, or specific steps in the participatory approach. The studies were then classified into four categories: (1) those that clearly stated the use of CBPR, CABs, CAGs, or co-design or described the participatory steps; (2) those that mentioned participatory approaches without providing detailed information; (3) those that did not mention any participatory approach; and (4) studies conducted entirely by community members.

### 2.6. Quality Assessment

The Mixed Method Appraisal Tool (MMAT, Version 2018) [38] was applied for a qualitative assessment of all included studies by two reviewers (CL and JL). The MMAT was suitable for this review due to its versatility in appraising different study designs, including quantitative, qualitative, and mixed methods. The tool uses criteria related to the clarity of the research questions, appropriateness of the study design, representativeness of the sample, outcome data’s completeness, adherence to the intervention, and interpretation of the results [38]. 

## 3. Results

### 3.1. Study Selection

The search across all five databases resulted in 1918 studies, of which 945 duplicates were excluded. After screening the title and abstract, 865 records were excluded, resulting in 108 articles for full-text review. Of these, 19 met the inclusion criteria. Two authors (CL and JL) independently conducted the screening process, and conflicts were resolved by discussion and consensus. 

### 3.2. Study Characteristics

Appendix B Table A2 presents the characteristics of the included studies.

#### 3.2.1. Study Design

All papers included in this review were published in English. Studies used qualitative methods (*n* = 5) [39,40,41,42,43], quasi-experimental methods (*n* = 4) [44,45,46,47], mixed methods (*n* = 4) [48,49,50,51], non-RCT (*n* = 3) [52,53,54], RCT (*n* = 2) [55,56], and cross-sectional methods (*n* = 1). Most studies (*n* = 12) were conducted in North America—USA, Canada, and Greenland, followed by studies in Oceania—the Federated States of Micronesia (*n* = 3), Asia—India and Japan (*n* = 2), South Africa (*n* = 1), and South America—Ecuador (*n* = 1). Studies were published between 2001 and 2023; nine were published between 2010–2019, seven were published after 2020, and three studies were published before 2010.

#### 3.2.2. Study Population and Sample

Sample sizes were generally small. Most studies (*n* = 11) had a sample smaller than 100 participants. The sample sizes ranged from 10 to 531; the mean was 132.9. All studies targeted First Nations populations and communities. Studies reported the participants’ ages differently, and some did not mention it (*n* = 5), which was a barrier to calculating the mean. The participants’ ages ranged from 6 months to 79 years old. Approximately half of studies (*n* = 10) had more than 50% female participants. However, six studies did not report gender.

#### 3.2.3. Method—Participatory Approach

Appendix C Table A3 shows whether the study used any participatory approach to develop, implement, and evaluate the intervention. Out of 19 studies, 12 explicitly used Community-Based Participatory Research (CBPR) or clearly outlined steps of a participatory approach, such as co-design, shared decision making, or active involvement of community members throughout the research/intervention process. A total of three studies cited the use of a participatory approach but lacked specific details on how the community or participants were involved in the research/intervention process. The other three studies did not describe any participatory approach or involvement of community members in the research process. One intervention was initiated, designed, and conducted by community members, with the study merely assessing or evaluating the outcomes of this community-led initiative.

#### 3.2.4. Quality Appraisal

The quality scores varied across the studies, with the lowest scoring study at 42.9% and the highest score at 100%, with an average of 77.3%. Overall, most studies (*n* = 12) received a high score—higher than 80%. However, two studies scored below 50%. The studies with low scores were due to a lack of details about sampling, statistical analyses, risk of bias, or the absence of some reported outcomes in the results (Appendix D Table A4). 

### 3.3. Interventions’ Characteristics and Main Outcomes

#### 3.3.1. Interventions’ Aims

Of the interventions, eight aimed to facilitate cultural reintegration, such as reconnecting with the traditional food system and increasing the intake of native foods [39,40,41,42,43,47,51,54]. Five interventions focused on improving diet and promoting healthy eating behaviours, including increasing FV intake and reducing unhealthy food consumption [46,47,48,49,53]. Four interventions aimed to enhance overall health and wellbeing [48,49,50,57], while another four aimed to improve access to healthy, local, and native foods [40,41,42,43], and four promoted physical activity [47,48,50,53]. Two interventions aimed to enhance food security or cultural food security [52,57]; two promoted dietary diversity and nutrient intake [44,52]; two focused on improving knowledge about nutrition, sustainability, and Indigenous foods [46,48]; and two supported local food production and consumption [49,50]. Furthermore, two targeted health promotion in specific conditions, such as diabetes and cardiovascular disease [55,56]. One intervention aimed to provide evidence of the importance of local food for food security and health [45]. Most interventions had multiple objectives, as detailed in Appendix B Table A2.

#### 3.3.2. Interventions’ Duration and Target Population

The duration of interventions varied widely from 1 month to 15 years. Most interventions (*n* = 13) lasted one year or more, which aligns with the participatory approach previously described. All interventions focused on First Nations populations and communities, as described in Appendix B Table A2. 

#### 3.3.3. Interventions’ Activities

**Figure 2:**
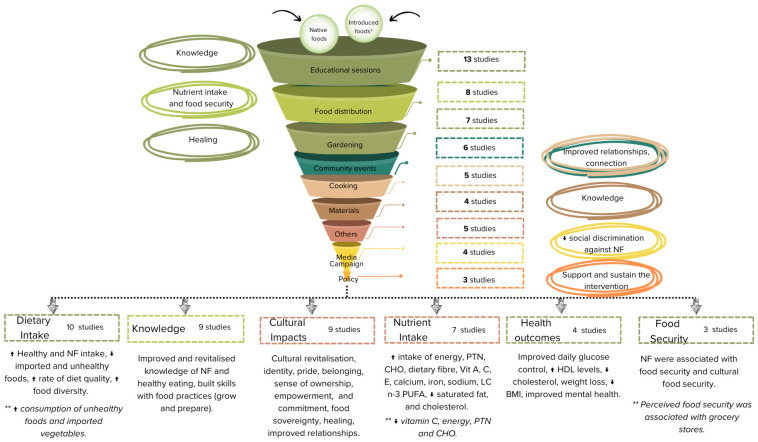
Distribution of intervention activities and main outcomes. * Introduced foods—non-native foods. ** Although studies showed improvements in various outcomes, they also reported mixed or unintended results, such as a reduction in specific nutrients (e.g., vitamin C) or associations of food security with grocery stores instead of native foods. NF—Native Foods; PTN—Protein; CHO—Carbohydrate; Vit—Vitamin; LC n-3 PUFA—Long-Chain Omega-3 Polyunsaturated Fatty Acid; HDL—High-Density Lipoprotein; BMI—Body Mass Index.

Appendix B Table A2 presents the detailed characteristics of the interventions, and Figure 2 shows a summary of the type of interventions. Most interventions (*n* = 16) combined native foods with other introduced healthy foods in their activities to achieve the previously outlined objectives. Interventions included a diverse combination of native foods, including animal-derived foods such as marine mammals and fish [44,53,54,55,56,57], as well as plant-based foods such as wild rice [48]; grains (kosayo—beans cooked with millet flour and berries) [39]; yams [44]; vegetables; and wild fruits such as perennial lily [39], yellow-fleshed banana [45,49], and breadfruit varieties [45,49,50]. Only three interventions focused exclusively on native foods in their activities, such as salmon [54,57] and some native greens and vegetables [51]. Overall, the majority of interventions (*n* = 15) integrated more than one category of activity. Two studies focused solely on activities related to food distribution [44,52], one study concentrated on educational sessions [46], and another focused exclusively on cooking activities [51]. 

Thirteen studies incorporated educational sessions as part of their interventions [40,41,42,43,45,46,47,48,49,50,53,54,57]. These sessions covered different topics, such as healthy eating [53]; food skills [43]; the benefits of native foods [49]; and traditional food preparation methods such as using a charcoal oven [50], gardening, and harvesting. Some schools included lessons on healthy eating [46], the benefits of traditional methods and native foods, and dietary diversification [46,49]. In some cases, elders passed on knowledge to youth, covering traditional food methods, cultural values, and Indigenous language [40,41,47]. 

Eight studies included food distribution as a strategy to enhance access to native foods [39,42,43,44,52,54,56,57]. For example, the Local Food to School (LF2S) program in Canada used local food pantries to distribute local foods to schools (e.g., meals with native foods) and other public organisations [42,43]. Similarly, the Nega Elicarvigmun (Fish-to-School) program in the USA served locally caught salmon weekly to students, aiming to reconnect them with their traditional food system and increase native food consumption [54]. 

Activities focusing on how to grow and prepare native foods were also included in the studies. Gardening activities were included in seven interventions [40,41,42,43,47,48,50]. These activities often took place in school gardens with native foods, where students and elders combined scientific knowledge with Indigenous knowledge, using respectful harvesting and traditional protocols [40,41,42,43,47]. Five studies included cooking activities designed to build the capacity to prepare healthy meals using native foods, including participatory cooking sessions and recipes focusing on native foods [39,49,50,51,53]. Community events such as fairs [53], celebration days [42,43,54], and communal gatherings [39,57] were also used as a form of intervention in six studies. 

To support knowledge transmission about native foods and traditional practices, four interventions also developed educational materials, such as guides, pamphlets, posters, recipe books, clipart, and storybooks [42,53,55,56]. Media campaigns were used in four studies to increase awareness and knowledge about native foods, including radio [53], newsletters and newspapers with recipes using native foods [39,53], posters [45], and the promotion of local foods through “Go Local” slogans [45,49]. For instance, Kaufer et al. [49] implemented the “Go local and Go Yellow” messages in some communities in Federated States of Micronesia to promote vitamin A-rich native foods. 

Three studies included local and regional policies to promote native foods. Blanchet et al., using a participatory approach, included community members to participate and decide on water management to facilitate the inclusion and reproduction of Okanagan Sockeye Salmon in Canada. Cueva et al. [40,41] implemented school guidelines to promote healthy foods and beverages. Other activities included in five studies involved counselling [55], environmental changes (e.g., constructing fish passages to support salmon migration) [57], labelling dishes with their traditional names [39], youth drama club, games, planting competitions, charcoal oven development [45], and seed and plant distribution [50]. 

#### 3.3.4. Outcomes 

The main outcomes reported by the studies included improvements in dietary and nutrient intake, increased knowledge, improved health, positive cultural impacts, and enhanced food security. 

##### Improvements in Dietary and Nutrient Intake

Dietary intake was analysed in 10 studies (52.6%) using Food Frequency Questionnaires (FFQs), 24-h recalls, or surveys. The improvements in dietary intake included increased native food consumption [45,49,50,51,53,54,55,56,57]; improved dietary diversity [45,49,51]; higher diet quality scores [54]; and reduced intake of unhealthy and less sustainable foods, such as imported meats, fruits, and added sugars [49,53,56]. Nine studies reported positive dietary outcomes, and eight studies incorporated educational sessions or materials in combination with other activities such as counselling, food distribution, cooking, community events, policy development, media campaigns, or gardening. One study [51] focused exclusively on cooking activities. 

Eight studies showed significant improvements in dietary outcomes, including increased healthy and native food intake, reduced imported and unhealthy food intake, and increased rates of diet quality and food diversity [45,49,50,51,53,54,55,57]. For example, Kaufer et al. [49] in the Federated States of Micronesia used workshops, cooking activities, and media campaigns to promote local food production and consumption. The intervention resulted in a 475% increase in the consumption frequency of giant swamp taro and a 53% increase in the consumption of local bananas (*p* < 0.01). Similarly, Roche et al. [51] in Ecuador conducted a 12-day participatory cooking session intervention focusing on Stinging nettle (*Urtica dioica*), round-leaved dock (*Rumex obtusifolius*), and other native vegetables, which led to higher dietary diversity scores in the intervention group (IG: 15.0 ± 4.4) compared to the control group (CG: 13.4 ± 4.6). Furthermore, the likelihood of mothers in the intervention group feeding their children native leafy greens was almost 10 times greater than in the control group (adjusted odds ratio (aOR): 9.5; 95% CI: 4.37, 20.21; *p* < 0.001). Two studies that also used educational sessions associated with other activities as part of their positive dietary outcomes also presented an increased consumption frequency of imported drinks with sugar and flour products [49] and imported vegetables [50]. One study that used educational sessions in combination with gardening activities presented low self-rated consumed food such as blueberries (3.00 = occasionally), raspberries (2.72 = a little/occasionally), and fish (2.72 = a little/occasionally) [48]. 

Seven studies (36.8%) analysed nutrient intake using FFQ [51,53,56] or 24-h recall [44,52] or a combination of both [45,49], and all reported improvements in some nutrients, including carbohydrates; protein; dietary fibre; vitamins A, C, and E; calcium; iron; sodium; and long-chain omega-3 fatty acids, along with reduced saturated fats and cholesterol [44,45,49,51,52,53,56]. However, three studies [44,51,56] did not provide statistical analyses. Nevertheless, some studies observed decreases in certain nutrients, such as reduced vitamin C [52], protein, and carbohydrates [53]. Reductions in carbohydrates, saturated fat, and cholesterol were associated with decreased consumption of unhealthy foods which were de-promoted by some studies [49,53]. Different strategies were used in the interventions presenting nutrient intake improvements, with most studies using educational materials or sessions associated with other activities. However, it is also important to highlight that, from seven studies with nutrient improvements, three studies used food distribution as an activity [44,52,56].

Englberger et al. [45], utilising a combined intervention with workshops, media campaigns, and youth activities such as youth drama clubs to promote yellow native foods rich in vitamin A, such as yellow-fleshed banana, giant swamp taro, and breadfruit, demonstrated a significant increase in provitamin A carotenoid intake from 227 μg/person to 475 μg/person (*p* = 0.02), attributed to the increase consumption of these promoted native foods. Kaufer et al. [49] also showed that yellow-fleshed bananas, giant swamp taro, and breadfruit combined with other native vegetables contributed to 36–98% of overall micronutrient intake, including 97% of vitamin C, 44% of calcium, 36% of iron, and an increased in beta-carotene equivalents (BCEs), among community members in Pohnpeian, FSM. In Ecuador, the “Corazon en Familia” program—a 12-day cooking session involving mothers and Elders—revealed that native foods such as nettle and dock contributed significantly to the daily recommended intake of nutrients for their children, accounting for 6.7% of iron, 27.5% of folate, and 25.5% of magnesium [51].

The Dali Food System, a 15-year community-led initiative in India that distributes different native foods, such as sorghum, millet, pulses, and wild fruits, reported statistically significant improvements among mothers in the intervention group for energy intake (11,189 ± 3335 kJ vs. 10,193 ± 3738 kJ; *p* = 0.04), protein intake (68.9 ± 22.6 g vs. 60.4 ± 23.8 g; *p* < 0.01), dietary fibre (40.8 ± 19.6 g vs. 32.5 ± 19.3 g; *p* < 0.01), and iron intake (15.8 ± 6.6 mg vs. 13.7 ± 9.1 mg; *p* < 0.01) [52]. However, a significant reduction in vitamin C intake was also observed (19.7 ± 35.5 mg vs. 21.7 ± 26.1 mg; *p* = 0.04) [52].

##### Health Outcomes

Four studies (21.1%) reported some health outcomes. Two studies presented statistically significant positive impacts [53,56], one study reported positive impacts without statistics, and one found no significant improvements [45]. The randomised controlled trial by Lewis et al. [56], conducted among Greenlandic native communities, compared a traditional marine diet (due to their high protein and fat content alongside low carbohydrate levels) with a Western diet through native salmon distribution and educational materials. The intervention group consuming a traditional diet with native salmon for four weeks showed improved daily glucose control (mean daily blood glucose decrease of 0.17 mmol/L-95% CI 0.05, 0.29; *p* = 0.006; maximum daily blood glucose decrease of 0.26 mmol/L-95% CI 0.06, 0.46; *p* = 0.010), increased HDL levels, reduced cholesterol ratio by 4% (95% CI 1, 9; *p* = 0.018), and reduced weight (average weight loss of 0.5 kg; 95% CI 0.09, 0.90; *p* = 0.016). Kolahdooz et al. [53], in Canada, used a multi-component intervention for 12 months (educational sessions, cooking activities, community events, and media campaigns) to increase the visibility and accessibility of healthy foods, including native ones, such as some marine animals and local berries. The authors reported a reduction in BMI among the intervention group. Participants in a 2-year qualitative study in Canada reported that gardening activities contributed to their mental health [43].

##### Positive Cultural Impacts

Nine studies (47.4%) used interviews, focus groups, photovoice, observations, and surveys to capture participants’ perceptions and feelings about the interventions, and all of them presented some positive cultural impacts [39,40,41,42,43,48,49,51,54]. Participants in studies involving media campaigns reported a reduction in social discrimination and more positive attitudes toward native foods [39,49]. Land-based activities such as gardening and gathering native foods in the forest were linked to wellbeing, life satisfaction, and healing from past trauma [42,43,48]. 

Participatory approaches and culturally appropriate activities such as community events celebrating Indigenous culture and foods [39,43,54], incorporation of Indigenous languages in dishes and activities [39,43,51], and garden and kitchen activities led by elders sharing traditional knowledge [40,41,42,43,51] were associated with enhanced identity, pride, belonging, sense of ownership, empowerment, commitment, food sovereignty, healing, connection with the community, and improved relationships. Participants in the “Feast for the Future” program in the USA expressed a strong sense of ownership and commitment due to the community participatory approach used in the intervention [41]. In the “Local Food to School” program in Canada, participants noted that gardening activities and educational sessions on land management and traditional food practices on land contributed to healing from past traumas [43]. Participants from the “Learning Garden Program” also reported improvements in health, life satisfaction, and community connectedness through activities like harvesting and locating edible berries in the forest [48].

##### Increased Knowledge

Nine studies (47.4%) that reported knowledge improvements used educational sessions. Of those, eight studies associated it with other activities, such as community events, gardening, cooking activities, and others. The outcomes included improved traditional food knowledge, increased awareness of healthy eating, and skills for growing and preparing food [41,42,43,47,48,49,50,54]. Kindergarten students in the USA’s “Veggies for kids” program demonstrated significant improvements (*p* < 0.01) in identifying asparagus, squash, lemon, spinach, and blueberry and showed an increased willingness to try squash after the intervention, which included educational sessions and gardening activities. 

##### Food Security

Three studies (15.8%) discussed food security outcomes. Two studies using food distribution in combination with other activities showed positive outcomes [42,57]. Blanchet et al. [57], in a cross-sectional study evaluating the Skaha Lake program in Canada, showed that 80.6% of participants considered traditional food important for household food security, and household access to salmon (*p* = 0.0216) and receiving salmon from a community member (*p* = 0.040) were significantly associated with cultural food security. McEachern et al. [42] highlighted that school food programs played a significant role in food security and sovereignty among participants, though without statistical support. However, Stroink and Nestor [48], using educational sessions with gardening activities, found that even though participants used their gardening skills to grow their own foods, perceived food security remained linked to the availability and affordability of food in grocery stores, rather than local food sources. Studies that incorporated decision making in local policies or guidelines aimed to support and sustain the interventions [40,41,57].

##### Challenges

All the studies underscored the benefits of incorporating native foods into community interventions. However, they also highlighted barriers such as limited access and knowledge about native foods due to the loss of family connection [57], challenges imposed by history and colonisation [41], seasonality, high cost, unreliable funding, food regulations that hinder the integration of native foods into current food systems [42], and a lack of space for cultivation [50]. 

## 4. Discussion

Findings from this scoping review highlight that native foods may play an important role in enhancing food security, nutrient intake, and promoting wellbeing in Indigenous communities. However, more details about their nutritional composition are needed to evaluate their real contributions to dietary quality. Except for three studies [45,49,55] that explicitly linked the nutritional composition of the native foods to the specific outcomes, such as vitamin A and omega-3 polyunsaturated fats, other studies did not provide detailed nutritional information about these foods, which limits the full assessment of their direct nutritional impacts. Moreover, studies reporting changes in nutrient intake applied different tools, such as FFQ and 24-h recalls, which can impact the consistency and comparability of the findings. 

Self-reported health outcomes, such as the improvement in mental health reported in one study [43], could benefit from the use of validated assessment tools in future research to enhance the reliability of the findings. Two studies [44,54] that scored lower than 50% on the quality appraisal may have had some biases in their specific results. However, their inclusion did not interfere with the overall results of this scoping review, as the findings were also supported by other more rigorous studies included in this review—improvement in micronutrient intake, diet quality, and community engagement.

Previous research has also shown significant improvements in dietary and nutrient intake resulting from multicomponent interventions using native foods. Redmon et al. [58] conducted a randomised controlled trial in Native American communities to prevent obesity, employing cooking demonstrations, taste testing in local food stores, school lessons on native foods and traditional food practices, educational materials, and media campaigns. These interventions aimed to encourage the consumption of healthy foods, including native ones, while discouraging the consumption of ultra-processed foods like sweetened breakfast cereals. The study found a greater decrease in the carbohydrate, total fat, saturated fat, and monounsaturated fat intake in the intervention group (*p* < 0.05). However, there was no statistically significant reduction in total sugar intake. Other studies have also highlighted the importance of native foods for micronutrient intake, including iron, calcium [59], vitamin A, and zinc [60]. Gagné et al. [61] evaluated the impact of native food consumption on the nutrient intake of preschool Inuit children in Nunavik. They observed that, despite low native food consumption, children who consumed native foods had significantly (*p* < 0.05) higher intakes of protein, iron, zinc, copper, phosphorus, selenium, riboflavin, vitamin B12, niacin, and pantothenic acid, alongside lower intakes of energy and carbohydrates. These findings support the potential of native foods to contribute to healthier dietary patterns due to their nutrient density [1,2].

Similarly to this review, other studies also reported the significance of native foods in enhancing food security within Indigenous communities [62,63,64,65,66,67]. McKerchar et al. [68] highlighted the importance of native foods in supplying and providing income to Maori people, which enhanced food security. Ferguson et al. [69] reported that 40% of the 76% of food-insecure individuals from remote Aboriginal communities in the Northern Territory, Australia, consumed native foods during these periods of food insecurity. However, the literature also highlights that access to native foods is one of the most significant barriers to food security for First Nations peoples, stemming from colonisation, cultural loss, environmental degradation, and negligent policies [70]. This lack of access of native foods, mainly in urban areas [71], can be one of the causes of the perceived food security being still strongly associated with the availability and affordability of foods in grocery stores, found in one of the studies in this review [48]. Canada’s Indigenous Food Bank program used a food distribution intervention to make fruits and vegetables, including native ones, more accessible to Indigenous people living in urban areas [72]. Participants noted the importance of this type of program for food security and empowerment through culturally appropriate foods [72].

Findings from this scoping review also showed that interventions using native foods, which positively impact nutrient intake, can be linked to preventing chronic diseases such as diabetes and cardiovascular diseases [56] and improving mental health [43]. Similarly, Birch et al. [73] and Pour et al. [74] also suggested that some Australian native grains may prevent non-communicable diseases such as diabetes and heart disease due to their nutritional composition and bioactive compounds, such as phenolic and polyunsaturated fatty acids. Additional studies also showed that some traditional food practices such as gathering, fishing, hunting, and eating native foods can improve mental health, as they foster cultural identity, nutritional health, and holistic well-being among First Nations peoples [75,76]. Studies show that native foods can also promote sustainable practices and foster cultural identity, enhancing communities’ health, healing, and resilience [66,67].

Most studies in this review used multicomponent strategies, combining educational sessions with hands-on activities such as gardening and cooking, food distribution, community engagement, and media campaigns to increase knowledge and achieve nutritional and health outcomes. Similarly, Gumelar and Tangpukdee [77] used video recordings and booklets focused on local and native foods in Indonesia and reported a significant increase in mothers’ knowledge and improvements in children’s anthropometry after the intervention (*p* < 0.05). Lee et al. [78] support the idea that multicomponent interventions are necessary to enhance nutrition and health outcomes among First Nations peoples, including economic strategies, food supplementation, food supply, and community-directed programs. Browne et al. [79] analysed previous programs addressing diet-related health outcomes for Aboriginal and Torres Strait Islander peoples. They found that multicomponent interventions involving community members in the development, implementation, and evaluation stages are more likely to succeed. 

All twelve studies using participatory methods in this review reported significant cultural impacts, such as cultural revitalisation, healing from past traumas, and improved relationships. Involving community members in all stages of the program or intervention—design, implementation, evaluation, and dissemination of results—fostered a sense of ownership, collaboration, respect, and reciprocity, leading to more sustainable nutritional and health outcomes [80,81]. Moreover, participatory methods can better address the community’s real needs through culturally relevant solutions [80,81].

Since 2007, the United Nations Declaration on the Rights of Indigenous Peoples (UNDRIP) has provided a framework to support the rights of First Nations peoples globally [82]. This foundational international human rights instrument emphasises Indigenous peoples’ rights to make decision in any research and policies that impact their lives [80,82,83,84]. Aligning programs and interventions with this framework allows these populations co-create knowledge and increase the effectiveness and relevance of policies and research [80].

In the context of integrating native foods into our food systems, particularly within Indigenous communities, several UNDRIP articles explicitly recognise Indigenous peoples’ rights in policies and research related to this topic [28]. *Article 24* affirms their *right to maintain their health systems and traditional plants [28]*. *Article 13* recognises *Indigenous rights to revitalise and transmit their knowledge, supporting and promoting traditional food practices*, including native foods [28]. These articles acknowledge the importance of traditional food practices among these communities in promoting health [28,80,84]. In terms of how it should be done, three other articles can guide research and policies regarding this topic. *Articles 4* and *18* recognise *Indigenous self-determination and the right to participate in any decision-making processes about their lives [28]*. *Article 26* asserts *Indigenous rights to access all traditionally owned resources*, such as land to manage sustainable and traditional practices and maintain food systems that align with their cultural values [28]. Finally, *Article 31* emphasises *the right of Indigenous peoples to control, protect, and develop their culture and traditional knowledge*, which protects the intellectual property of Indigenous food-related knowledge and practices, ensuring that research and policies benefit communities while preserving their cultural integrity [28,84].

While all studies in this review aligned in some way with the UNDRIP by integrating native foods into communities’ diets, not all clearly stated the participatory approach used to ensure Indigenous self-determination and sovereignty. Barriers mentioned in the studies, such as loss of cultural connections and knowledge about these foods and lack of space to grow their foods, remain significant challenges to ensure more ethical integrity in programs and policies using native foods. 

## 5. Strengths and Limitations

This review offers a comprehensive understanding of the diverse ways in which native foods have been integrated into health and nutrition interventions, showing their potential to improve health, enhance cultural identity, and address food security issues within Indigenous communities. This broad scope allows for a deeper understanding of the flexibility and adaptability of multicomponent interventions and their contributions to some specific outcomes, providing valuable guidance for future programs and policy development.

Our scoping review has highlighted some gaps in the existing literature that require further investigation. These include the need for more detailed information on the nutritional composition of native foods to better understand their contributions to nutrient intake improvements. Additionally, future research should aim to establish clearer links between specific activities within interventions and their outcomes. Addressing these gaps will provide a more comprehensive approach to how native foods can be effectively utilised to improve health outcomes, particularly in Indigenous communities. For practice, our scoping review offers valuable insights for health promotion officers and policymakers, for example, regarding the importance of media campaigns to reduce discrimination against these foods, food distribution to address food security, and culturally safe approaches for health promotion interventions involving Indigenous communities. 

Nevertheless, the wide range of strategies included in the review also presented a significant limitation. The broad variety of activities, diverse outcomes, and study designs reported made it challenging to directly and statistically link specific strategies to specific outcomes. Therefore, the individual contribution of each activity to observed outcomes could not be clearly established. This complexity made it impractical to conduct a more detailed systematic review or meta-analysis, which would have provided a clearer understanding of the effectiveness of each strategy. Future research should consider focusing on more targeted interventions and utilising standardised outcome measures to better evaluate which specific activities lead to outcomes.

While every effort was made to conduct a comprehensive search, we may have missed some relevant studies, particularly those published in other languages or indexed in other databases. 

## 6. Conclusions

The findings from this review show the potential of multicomponent interventions using native foods to support dietary improvements, health outcomes, and cultural revitalisation within Indigenous communities. Additionally, culturally safe interventions that actively involved elders and community members in all steps of the program foster food sovereignty and community empowerment. We acknowledge that the nutrient-rich properties of native foods are also attributed to their minimal processing, rather than solely to their origin. Moreover, it is important to note that the urban lifestyle, such as social interactions and physical activity levels, has profoundly impacted Indigenous health, highlighting the need for future research to explore its association with the consumption of native foods and its impacts. However, despite the reported benefits, significant barriers such as the loss of traditional knowledge, limited access to native foods, and lack of policy support continue being significant challenges. Aligning future programs with the United Nations Declaration on the Rights of Indigenous Peoples (UNDRIP) can promote greater Indigenous self-determination and sovereignty in food practices. Interventions must prioritise participatory approaches, ensuring Indigenous voices guide the design, implementation, and evaluation stages.

## Appendix A. Search Strategy

**Table A1:** Search strategy employed in the review.

Search Terms	Scopus	Embase	Web of Science Core Collection	Medline	PubMed
(“native food” OR “bush food” OR “indigenous food” OR “aboriginal food” OR “wild edible foods” OR “traditional food”) AND (“health promotion” OR “nutrition intervention” OR “nutrition education” OR “health intervention” OR “health education” OR “intervention” OR “food-based intervention” OR “gardening” OR “garden” OR “urban garden”)	590	449	378	376	125
Limits - Publication date = no restrictions - Publication types = peer-reviewed scientific papers only - Languages = English and Portuguese - Study participants = human studies only - Intervention = use of native foods to promote or support health or address food insecurity; all types of interventions using native foods, such as cooking, gardening, nutrition education, etc. Comparator = no use of native foods, pre- and post-intervention, or no comparison					

## Appendix B. Characteristics of Included Individual Studies

**Table A2:** Characteristics of included individual studies (*n* = 19).

Author(s), Year, Country	Sample Characteristics: Sample Size (IG:CG); Mean Age (Years) (IG:CG); % Female	Target Population	Intervention (Name—If Applicable, Aim, Duration, Type of Activities, Activity Details)	Native Food	Outcomes	Results
**Randomised Controlled Trial (RCT)**						
Oppezzo et al., 2022, USA [55].	299 (151;148); 46.3 years; 48.5% females.	Alaska native adults with high blood pressure or high cholesterol smoking daily and residing in the Norton Sound region of Alaska.	Healing and empowering Alaskan lives toward healthy hearts. Promoting heart health. 12 months. Counselling (30 min—baseline, 3 mo, 6 mo, and 12 mo) and educational materials. IG: medication adherence + Alaska Native diet (foods rich in omega-3 polyunsaturated fats from marine mammals and fish). CG: tobacco + PA: focused on tobacco cessation strategies and increasing PA levels. Both received information about importance of medication adherence. Materials: culturally tailored cookbook with heart-healthy recipes and a bag to help to organise medications; traditional Alaska Native values such as respect for elders, land, and family; featured photos of traditional foods, land, and people.	Foods rich in heart-healthy omega-3 polyunsaturated fats from marine mammals and fish.	Dietary Intake: 34-item FFQ adapted for Alaska Native communities—ratios of heart-healthy foods and traditional heart-healthy foods consumed. Medication adherence: two questions.	Dietary intake: IG ↑ heart-healthy foods (*p* = 0.014) at 6 months and traditional heart-healthy foods ration (aged 47 and older) (*p* = 0.031). Medication adherence: Both groups reported high levels of adherence.
Lewis et al., 2023, Greenland [56].	56 (27;29) 56 years; 54% female.	Greenlandic Inuit-Nuuk population, Qasigiannguit and Qaanaaq (adults)	Investigate the effects of traditional marine diets versus Western diets on glucose homeostasis and cardio-metabolic health in individuals with and without the TBC1D4 p.Arg684Ter variant (associated with insulin resistance and increased risk of type 2 DM). 4 weeks. Food distribution and educational materials. TD—marine-based, high in PTN and fat (over 40%) and low in CHO (<30%); WD—high in imported meats, breads, pasta, rice, and cereals; high carb content (55–65% of energy intake); and moderate fat content (30–35%). A proportion of the diet was provided to participants (>20%), and the remaining participants were provided with basic written instructions to follow the specific diet.	Seal meat, whale blubber, salmon, trout, halibut, shrimps, red fish, cod, catfish, other fish.	Dietary intake (FFQ); glucose homeostasis (fasting, oral glucose tolerance test and continuous glucose monitoring); cardiometabolic markers (total cholesterol, LDL, HDL, and TAG systolic and diastolic blood pressure; waist circumference; weight; and body fat).	Dietary intake: ↑ total marine mammals consumption (TD from 15 g/day to 26 g/day, ↓ to 1g/day with the WD), ↓ imported meat and sweetened drink intake and added sugar. Imported meat–WD: ↑ 15g/day; TD ↓ 38 g/day, ↓ sweetened drink intake from 228 g/day to 119 g/day, and added sugar from 12 g/day to 2 g/day. Nutrient intake: TD ↑ intake of long-chain n-3 polyunsaturated fatty acids: 4·6 (95% CI 3·0; 6·2) %-point higher after the traditional period (*p* < 0·001) Health outcomes: Glucose homeostasis: TD significantly ↓ mean daily blood glucose by 0.17 mmol/L (95% CI 0.05, 0.29; *p* = 0.006) and maximum daily blood glucose by 0.26 mmol/L (95% CI 0.06, 0.46; *p* = 0.010) compared to the WD. Cardiometabolic markers: TD: an average weight loss of 0.5 kg (95% CI 0.09, 0.90; *p* = 0.016), ↓ cholesterol ratio by 4% (95% CI 1, 9; *p* = 0.018) and HDL cholesterol 0.09 mmol/L (95% CI 0.03, 0.15; *p* = 0.006) after adjustment for weight loss.
**Non-Randomised Controlled Trial (CT)**						
Schmid et al., 2007, India [52].	Mothers: 220 (124;96); 24.5 years; 24.0 years; 100% female. Children: 220 (124;96); 6–39 months.	Dalit mothers and their children in rural Andhra Pradesh, India.	The traditional Dalit food system (a community-led program). Enhance dietary diversity and nutrient intake among Dalit mothers and their children in 19 villages, focusing on sustainable agriculture and food security. 15 years. Food distribution. Local production, storage, and distribution of native and introduced foods (800,000 kg/year sorghum, which was sold at low prices during scarcity/rainy season).	Sorghum, millet, pulses, wild fruits, roots, tubers, uncultivated native green leafy vegetables.	Nutrient intake: 24-hour recalls (summer and rainy seasons) compared to Indian RDI for women.	Nutrient intake: Mothers from IG had significantly higher intakes of energy (mean ± SD: 12,218 ± 3511 kJ vs. 11,155 ± 3347 kJ; *p* = 0.02), PTN (77.5 ± 25.1 g vs. 71.1 ± 25.2 g; *p* = 0.05), and dietary fibre (48.5 ± 23.2 g vs. 42.0 ± 23.01 g; *p* = 0.03) in summer. During the rainy season, mothers from IG had significantly higher intakes of energy (11,189 ± 3335 kJ vs. 10,193 ± 3738 kJ; *p* = 0.04), PTN (68.9 ± 22.6 g vs. 60.4 ± 23.8 g; *p* < 0.01), dietary fibre (40.8 ± 19.6 g vs. 32.5 ± 19.3 g; *p* < 0.01), and iron (15.8 ± 6.6 mg vs. 13.7 ± 9.1 mg; *p* < 0.01). However, they had significantly lower intake of vitamin C (19.7 ± 35.5 mg vs. 21.7 ± 26.1 mg; *p* = 0.04). No differences were found in children’s intakes. During both seasons, mothers from IG and CG had mean energy and PTN intakes above, and median iron and vitamins A and C intakes below recommendations. Sorghum contributed to 56% of total iron, 31% of total energy, and 35% of total PTN intake in IG. Green leafy vegetables contributed to 2% of total iron and 11% of total vitamin C intake in IG, three times lower than CG. Fruits and vegetables: major source of vitamin A (summer: 54% of vitamin A intake in IG, 40% in CG. Rainy season: uncultivated green leafy vegetables—43% of vitamin A intake in IG, 36% in CG). Millet was consumed by <1% of women in both groups.
Kolahdooz et al., 2014, Canada [53].	332 (221:111); 45.5 years and 41.9 years; 80–82% female.	Food shoppers and preparers in Inuit and Inuvialuit communities in northern Canada.	“Healthy Foods North” (HFN). Improve diet and increase PA. 12 months. Educational sessions, cooking activities, educational materials, media campaigns, and community events. Activities to increase the availability, accessibility, and visibility of healthy foods and opportunities for PA and de-promote unhealthy foods, e.g., workshops on PA and healthy eating, cooking with TF and market foods, posters, recipe books, newsletters, health fairs. IG: two remote communities received the intervention. CG: two remote communities received a delayed intervention (after data collection).	Caribou, Arctic char, seal, local fish, Muktuk (whale skin and blubber) ptarmigan, goose, and local berries.	Dietary intake: A culturally appropriate validated FFQ to assess consumption of de-promoted and promoted foods and an Adult Impact Questionnaire (AIQ) to determine food acquisition and preparation behaviours.	Dietary intake: ↑ TF intake from 1.4 to 1.7 times/day (IG); ↓ some de-promoted food intake in the IG compared to CG: unhealthy drinks (IG: ↓ 166.6 g/day, CG: ↑ 21.9 g/day, *p* ≤ 0.05), high-fat dairy products (IG: from 19 to 11 g/day, CG: from 6 to 18 g/day, *p* ≤ 0.05), and high-fat meats (IG: from 46 to 27 g/day, CG: from 24 to 33 g/day, *p* ≤ 0.001). Nutrient intake: IG: ↓ of 317 kcal/day, ↓ protein intake (21 g/day), carbohydrate intake (37g/day). Health outcomes: ↓ BMI (*p* = 0.002). Others: ↑ healthy methods to prepare foods, such as microwaving without fat, pan frying in own fat, and drained (IG: from 3.9 to 4.3 times/day, CG: from 4.5 to 4.3, *p* ≤ 0.001).
Bersamin et al., 2019, USA [54].	76 (38:38); 13.9 years and 14.3 years; 55% female.	Yup’ik middle and high school students in two rural, remote Alaska Native communities.	Nega Elicarvigmun (Fish-to-School) program. Reconnect students with their TFS and increase TF intake. 9 months. Food distribution, educational sessions, and community events. Cafeteria served locally caught salmon weekly. Classroom taught five cultural lessons on benefits of local and traditional foods. Community: four intergenerational community events were organised to celebrate traditional foods, where students prepared the foods. IG: received all intervention components. CG: to ensure community benefits, CG also received salmon, but received the classroom lessons after the 4-month data collection (baseline, 4 months, and 9 months).	Focused on salmon, a central food in the Yup’ik diet.	Dietary intake: 24 hr recall using the Healthy Eating Index-2010 score. Fish intake: biomarker of fish and marine mammal intake. Attitudes and perceptions of the benefits of salmon, traditional food practices, and the impacts of food choices on the environment using a survey.	Dietary intake: IG: ↑ rate of diet quality 4.57 times greater (beta = 4.57; *p* < 0.05), ↑ fish intake 0.16 times more than the IG (beta = 0.16; *p* < 0.05). Perceptions: Community events promoted family and community engagement. Both improved beliefs and knowledge of importance of TF.
**Cross Sectional Studies**						
Blanchet et al., 2022, Canada [57].	257; 49.8 years; 70.2% female.	Syilx Okanagan Nation communities.	Skaha Lake program. Enhance cultural food security and health. 12 years. Food distribution, educational sessions, community events, policy, and environmental changes. Harvest to early childhood programs, school and health programs (in 2016 allowed a distribution of >13 000 kg). Lessons—raising salmon fry in the classroom, salmon gatherings, ceremonies, feasts, songs, prayers, Nsyilxcen language transmission, participation in water management and regional decisions, fish passage to support migration, fish releases.	Okanagan Sockeye Salmon.	Seasonal traditional salmon food consumption: TFFQ Cultural Food Security (CFS) Status. The 18-item USDA Household Food Security Survey Module (HFSSM) was adapted to Indigenous populations in Canada. Importance of cultural food security: close-ended questionnaire. Accessibility and barriers to eating salmon from survey.	Dietary intake: Improved salmon consumption and access: 85.6% ate salmon about twice per month in the last year, and 48.6% ate specifically Okanagan sockeye salmon. There was a positive association between the number of ways households accessed salmon and the frequency of consumption (r = 0.51727, *p* < 0.0001): 49.8% received salmon from a community program, 27.2% harvested salmon, 10.9% bought salmon from a store, 9.4% accessed fishing equipment provided by the program, 12.5% reported not benefiting from the intervention. Cultural food security (CFS): 80.6% of participants considered TF important for their household’s food security; 63.1% of households experienced cultural food insecurity (20.9% often worried and 42.2% sometimes worried about TF running out). Households’ overall access to salmon (*p* = 0.0216) and receiving salmon from a community member (*p* = 0.0403) were associated with CFS status. CFS status was positively associated with income-related food security status (*p*= 0.0115) and with perceived importance of CFS (*p* < 0.0001). Income-related food security: 46.5% of households experienced income-related food insecurity with varying degrees of severity (13.4% severe). Main barriers to salmon consumption: lack of access and availability, time constraints, lack of resource and knowledge, and loss of family connection.
**Qualitative Studies**						
Iwasaki-Goodman, 2017, Japan [39].	10; 6 mothers age 50s and 4 daughters age 30s; 100% female.	Ainu mothers and daughters in the Saru region of Biratori, Japan.	Improve the socio-cultural environment through reintroducing traditional Ainu food. ~10 years. Food distribution, cooking activities, media campaigns, community events, and labelling dishes with their traditional names. A core group of women in the local Ainu cultural preservation group was responsible for food preparation at community events. Monthly community newsletter with traditional Ainu food recipes, Ainu cooking classes, activities collecting wild plants, and ten communal gatherings annually attracting up to 300 participants. Ainu and non-Ainu members provided traditional dishes such as tonoto and inakibi gohan.	Many traditional Ainu foods. Wild plants: Turep (perennial lily), Pukusakina (anemone), and Noya (mugwort); meat and fish: Yu kama (deer meat) and Sipe (salmon); grains: Kosayo (beans cooked with millet flour and berries grown on amur cork).	Transmission of traditional Ainu food knowledge, perceptions of cultural identity, and changes in the society and environment. Observation of community events to understand the preparation and sharing of traditional Ainu dishes and the social dynamics involved. Interviews (after 10 years of the intervention)	Perceptions: The intervention provided a cultural revitalisation, cultural identity, and pride; reduced social discrimination; improved integration of wild plants and traditional methods of the Ainu and non-Ainu diet, which were once stigmatised and avoided; and increased awareness of environmental degradation. The intervention contributed to both Ainu and non-Ainu people participating in community events sharing traditional Ainu dishes, which supported cultural exchange. Traditional Ainu foods were used as a symbol of their heritage, balancing social power dynamics within the mainstream Japanese society.
Cueva et al., 2020, USA [41].	43 (15 farmers, 14 elders, and 16 CAB members). Average age: elders: 62; farmers: 42; and CAB members: 38. 63% female.	American Indian communities in three rural Indigenous areas in the Southwest US.	“Feast for the Future” (FFF). Promote access to healthy foods and revitalise TFS. ~3 years. Garden activities, educational sessions, and policy. Edible School Garden (24-week school year): gardening and nutrition curriculum for third–fifth graders integrating science and Indigenous knowledge. Traditional Foodways Education Program (youth ages 5–18, 24 sessions): transferring traditional food-based knowledge from elders and farmers to youth, including activities in community gardens teaching traditional planting, harvesting, food processing, local languages, and cultural stories. Policy and systems support: development of school wellness policies, such as guidelines for healthy foods and drinks.	The program promoted Indigenous foods and traditional food practices. No specific native foods were detailed.	Changes in community food access, cultural connections, and personal behaviour assessed via in-depth interviews.	Perceptions: The program facilitated a sense of belonging and cultural identity. The program also influenced families’ healthier choices, increasing access to healthy and homegrown TF and becoming more physically active. The use of a community-based participatory approach fostered a sense of ownership and commitment among the participants. Knowledge: Revitalising traditional farming and knowledge about health benefits of TF. Barriers: The challenges imposed by history and colonisation but also individual factors that impact food choices.
Cueva et al., 2020, USA [40].	44 American Indian students (fourth and fifth-grade); 9–11 years old, 48% female.				Perspectives of youth about the program via Photovoice.	Perceptions: Students expressed pride in knowing how to grow TF similar to their ancestors. Participants described TF as healthier than conventional foods, highlighting chili peppers, corn, and squash. The program supported positive connections to culture, community, and elders. Students expressed the importance of future generations learning about respecting and cultivating the land.
McEachern et al., 2022, Canada [42].	20; age and gender not specified.	Haida Gwaii communities (British Columbia)–school-aged youth and adolescents, Elders, teachers, farmers, hunters, and other stakeholders who are involved in the food system and cultural knowledge sharing.	The Learning Circles: Local Food to School (LF2S). Enhance access to local, healthy, and TF in school communities. ~2 years. Food distribution, educational sessions, educational materials, garden activities, and community events. Local food pantries: hubs for year-round access to local foods (distributed to schools and other public organisations), supporting food literacy and minimising food waste. School meal programs, such as weekly salad bars with locally grown and TF, breakfast smoothie programs using local berries, gardening projects (elders provided guidance on respectful harvesting and traditional protocols), and food skills workshops. Community-engaging events to promote local and TF, involving students, parents, and community members. A Haida-language resource was created in schools to support curriculum development (poster and book component).	Not specified.	Participants’ perspectives on the program: semi-structured interviews, photographs, activity tracking reports, newspaper articles, and meeting reports.	Perceptions: Increased access to TF, which contributed to community’s pride in their culture and identity. Fostered relationships, community engagement, and sustainable and culturally important food practices. It also positively impacted children’s dietary habits and health. Transition to Haida leadership and culturally rooted practices fostered a sense of empowerment and ownership and promoted food sovereignty. Knowledge: The program facilitated the growth of hands-on learning experiences, traditional practice, and food literacy within the community.
McEachern et al., 2022, Canada [43].	12; age and gender not specified.	Community members in Hazelton/Upper Skeena, located in northern British Columbia.	The Learning Circles: Local Food to School (LF2S). Enhance access to local, healthy, and TF in remote Indigenous school communities. ~2 years. Food distribution, educational sessions, garden activities, and community events. Youth trips to community gardens, support greenhouses and smokehouses, hosting Indigenous Peoples’ Celebration Days, food security by delivering $8000 of organic produce, connecting youth with the river and culture, developing community gardens, running programs focusing on food skills and food preservation, incorporating wild foods into children’s snacks, land-based education incorporating Gitxsenimx language, and medicinal plants.	Not specified.	Participant’s perspectives on the program: semi-directed interviews, process reporting, photographs and video footage, and photovoice.	Perceptions: Increased access to local and TF among school-aged youth. Partnerships facilitated community engagement and trust building among community members. Traditional food practices and the connection to the land contributed to healing from past traumas. Health: Gardening programs contributed to students’ mental health, such as a memory garden that helped students with grief. Food security: Schools’ food programs played a key role in food security and sovereignty. Knowledge: Knowledge sharing and increased understanding of nutrition, traditional food practices, and history of Indigenous peoples. Barriers: Higher cost of local foods, seasonality, unreliable funding for food-to-school programs, and food safety regulations that posed challenges to serving TF in schools.
**Quasi-Experimental Design (Pre–Post Intervention)**						
Leslie, 2001, USA [44].	16; ages ranged from 22 to 64; 25% female.	Native Hawaiians (adults).	Uli’eo Koa Program. Assure adequate intake of nutrient demands of an intense physical activity regimen. 11 weeks. Food distribution. It included PA and meals with a mix of native and introduced foods. The program had three phases. Phase I (3 weeks): daily exercise and three daily meals and snacks provided, including native foods. Breakfast and dinner were buffet style, and lunch and snacks were pre-packed to take away daily. An hour of light PA pre breakfast and an hour of intense PA before dinner. Phase II (8 weeks): two evening meals/week and exercise once a day three times/week. Phase III: final program assessment. The program provided large quantities of fish, very little chicken, and no high-fat red meat.	Taro, sweet potato, breadfruit, yams, leaves, seaweed, berries, banana, mountain apple, fish, shellfish, chicken, coconut, milk.	Nutritional analysis: 24-h recall (pre–post intervention) to measure changes over 11-week period and compare to the National Research Council (NRC) recommendations for dietary intake for adults.	Nutrient intake: ↑ Kj: 97–107%; ↑ PT 141–219% (↑ milk consumption); ↑ CHO: 89–99% (↑ sweet potato, poi, taro, whole wheat bread, brown rice, cereals); ↓ saturated fat: 114–83%, ↑ monounsaturated fat 102–110% and ↓ cholesterol 83–82%; ↑ vitamin A 175–326%, ↑ vitamin C 135%-517% (↑ FV consumption); ↑ vitamin E 73–124% (↑ cereals and whole wheat bread); ↑ calcium 59–120% (↑ milk); ↑ iron 140–204% (↑ food fortification breads, cereals, whole grains); ↑ sodium 128–147% (sodium-based preservatives in milk, breads, and cereals).
Englberger et al., 2010, Federated States of Micronesia [45].	26 for 24-hour recalls and 40 for 7-day FFQ; ages not specified; 100% female + one child per household (ages 1–10 years).	Females and children in Mand Community, Pohnpei, FSM.	Global health project. Provide evidence that local resources are critical for food security, nutrition and health. 2 years. Media campaigns, educational sessions, and others. Youth drama club, games, mass media promotions (“Go local” slogan), poster campaigns, planting competitions, charcoal oven development, schoolroom activities, and agriculture and cooking workshops.	Yellow-fleshed banana, giant swamp taro, and breadfruit varieties (chosen due to their provitamin A carotenoids and other nutrients content).	Nutrient intake, calories, vit. A and C: 24 h dietary recalls. Dietary diversity and TF consumption: 7-day FFQ. Attitudes and awareness towards TF and intervention activities. Anthropometric and health measurements: BMI, waist circumference, fasting plasma glucose, blood pressure.	Dietary intake: ↓ rice consumption from 846g/person to 544g/person (*p* = 0.0002). Dietary diversity and TF consumption: ↑ 53% of local banana, 475% of giant swamp taro, and 130% of local vegetables; ↑ number of yellow-fleshed banana varieties consumed from three to seven, ↑ food diversity from 4.8 local food groups to 5.5. Nutrient intake: ↑ provitamin A carotenoid intake from 227 μg/person to 475 μg/person (*p* = 0.02). Health outcomes: No significant improvements. Others: ↓ money spent on food from 63% of their monthly salary to 33%.
Mbhatsani et al., 2017, South Africa [46].	154; ages ranged from 9 to 14; gender not specified.	Primary school children in rural Vhembe District, Limpopo Province, South Africa.	Nutrition education on dietary diversification. Improve nutrition knowledge of Indigenous foods and change eating behaviour. 6 months. Educational sessions. 45–60 min lessons: (1) Healthy eating habits using the South African food-based dietary guidelines. Food cards, models, and roleplaying were used as demonstrations. (2) Dietary diversification using Indigenous foods: children selected some food combinations. (3) Health benefits of Indigenous food: children collected some wild fruits and brought a cooked Indigenous dish to the school.	Traditional foods available such as Dospyros mespiliformis Hoechst, Vangueria infestusta Burch, Berchemia discolor, boabab, careissa edulis Vahl, and Euclea divinorum Hierna.	Nutrition and Indigenous food knowledge: questionnaire, oral questions, and quizzes. Dietary intake: knowledge, availability, and consumption (KAC) questionnaire and a seven-day non-quantifiable FFQ.	Knowledge: Improvement in children’s knowledge and understanding of dietary diversification, healthy foods, and the benefits of Indigenous foods. Healthy eating knowledge (pre–post intervention correct answers): understanding of healthy eating from 40% to 83%, knowledge of nutrients from food groups 35%-NA, importance of breakfast 38%-NA, identification of carbohydrate-rich foods NA-80%, knowledge of combinations with starchy foods NA-85%, understanding of protein function NA-60%, recommended number of meals/day NA–75%. Dietary diversification: identification of starchy foods 35%-NA, knowledge of nutrients in fruits and vegetables 25%-NA, knowledge of legumes 85%-NA, importance of having a diverse diet 27.5%-NA, identification of food combinations/meal NA–90%. Benefits of Indigenous foods: knowledge of Indigenous food 82.55%-NA, identification of Indigenous fruits and vegetables 80%-NA, importance of eating fruits and vegetables 47.5–92.5%, sources of fruits and vegetables NA-82.5%, prevention of diseases through consumption of Indigenous fruits and vegetables NA–85%.
Emm et al. 2019, USA [47]	45 American Indian kindergarten students (on-reservation) and 486 (off-reservation); age and gender not specified.	Kindergarten students from American Indian communities and nearby rural areas in Nevada.	Veggies for kids. Increase FV intake, promote healthy beverages, PA, and cultural integration among the kindergarten students. 12 weeks. Educational sessions and gardening activities. MyPlate Education: teaching the students to identify food groups; FV identification and tasting; gardening experiences—learning how to grow their own vegetables, with an emphasis on traditional native foods; and tribal elders discussing TF and their significance, language, harvest, and use.	Wild onions, buck berries, pine nuts, and other native plants.	Knowledge about food groups, FV identification and willingness to try, water consumption, and PA survey.	Knowledge: Significant improvements in students’ knowledge of healthy eating, with higher post-test scores for identifying food groups. Recognition of USDA’s MyPlate (*p* < 0.001): off-reservation increased from 38% to 91%; on-reservation increased from 47% to 87%. Correct naming food groups: off-reservation (*p* < 0.001) from 0.8–4.0% to 51–74%; on-reservation from 0–4% to 62–82%. FV identification and willingness to try: off-reservation, of the 12 questions, all except two (lemon and strawberry) had statistically significant improvements (*p* < 0.001). On-reservation, statistically significant improvements (*p* < 0.01) in identifying asparagus, squash, lemon, spinach, and blueberry and willingness to try squash. Water consumption: off-reservation, selection of 16+ drinks/day increased from 14% to 26%; on-reservation increased from 11% to 20%.
**Mixed Methods**						
Stroink and Nelson, 2009, Canada [48].	Quantitative sample: 20; ages ranged from 15 to 66; 70% female. Qualitative sample: 6 adults and 12 children (Aroland) and 12 adults and 17 secondary students (Ginoogaming).	Adults and children in the Ginoogaming First Nation and Aroland First Nation communities in northwestern Ontario, Canada.	Learning garden program (place-based learning). Strengthen the knowledge for a sustainable local food system, improve nutrition, increase PA, and enhance health and wellbeing. 1 month. Educational sessions and gardening activities. The program connected the community members with the local bioregion and the history and culture of the community. Twice monthly workshops that combined practical gardening activities (soil preparation, composting, weeding and harvesting, and forest garden harvesting—locating edible berries and wild rice in the forest) and kitchen workshops (preparation of healthy meals and snacks) with discussions on cultural values, food, and health.	Forest foods: edible berries, wild rice, and various cultivated garden vegetables.	Quantitative data (survey after the intervention): perceived physical health and life satisfaction/wellbeing; self-rating of foods consumed; self-rated knowledge of how to access food from fishing, hunting, gathering, and cultivated gardening; food values considered to guide their food choices; perceived food security; perceived trust and connectedness within the community; and identification with Aboriginal culture. Qualitative data: observation and audio recordings in the workshops discussions.	Dietary intake: Most self-rated consumed foods were chicken (3.72 = occasionally/often), apples (3.50 = occasionally/often), bananas (3.39 = occasionally/often), and beef (3.39 = occasionally/often). Blueberries (3.00= occasionaly), raspberries (2.72 = a little/occasionally), and fish (2.72 = a little/occasionally) had lower ratings. Perceptions: Engaging in forest food activities was correlated with self-reported health, life satisfaction, and connectedness with the community. Knowledge: Participants used the gardening skills to plant gardens in their communities. They were more knowledgeable on locating edible berries (mean rating 2.63 on a 5-point scale), moderately knowledgeable on cultivated vegetable gardening (2.38), and least knowledgeable on locating places where wild rice grows. Food security: Perceived food security was strongly linked to the availability, affordability, and convenience of grocery stores rather than local food sources, showing the dominance of the global food system. The mean rating of how they accessed food was 4.25 (often) for “nearby grocery stores”.
Kaufer et al., 2010, Federated States of Micronesia [49].	26 for 24-hour recalls and 40 for FFQ; qualitative evaluation: 42 households; ages and gender not specified.	Youth and women, and some activities entire community in Pohnpeian.	Traditional food for health. Promote local food production and consumption to improve nutrition and health. 2 years. Educational sessions, cooking activities, and media campaigns. Education and training focusing on the benefits of local foods. Workshops (twice monthly, each 4–6 h) covering topics such as soil preparation, gardening, forest food harvesting, cultural values, and kitchen workshops. Social marketing: “Go local” and “Go Yellow” messages to promote local and vitamin A-rich foods such as giant swamp taro, yellow-fleshed banana, breadfruit, and green leafy vegetables.	Yellow-fleshed bananas, giant swamp taro, breadfruit, green leafy vegetables, local fruits (e.g., pineapple, papaya, guava, citrus), and local fish.	Quantitative data (pre–post intervention): dietary assessment: a seven-day FFQ and two non-consecutive 24-h recalls. Qualitative data (post-intervention): questionnaire and observations to analyse awareness, exposure, and attitudes toward the intervention.	Dietary intake: Consumption frequency: ↑ giant swamp taro (475%) and local bananas (53%) (*p* < 0.01), ↓ average daily rice from 847g to 544g (*p* < 0.008), ↑ local vegetables (130%) and fruits (*p* < 0.01), ↓ imported fruit (*p*< 0.01), ↑ drinks (coconut, madeu, lemon grass tea) and snacks (sugar cane, coconut husk, chestnuts) (*p* = 0.01), ↓ sugar from 3 days/week to 2 days/week (*p* < 0.01). However, ↑ consumption frequency of imported drinks with sugar and chicken eggs, liver, and imported meat. ↑ Dietary diversity (food group score, species diversity score, and food variety score) (*p* ≤ 0.04). However, ↑ consumption frequency of flour products (*p* < 0.01). Nutrient intake: ↓ energy intake by 11% (*p* = 0.04), ↓ CHO intake by 14% (*p* = 0.03), ↑ beta-carotene equivalents (BCEs) intake by 110% (*p* = 0.02). Local foods contributed 36–98% of all micronutrient intakes (97% of vitamin C, 44% of calcium, and 36% of iron intake). Perceptions: High awareness and participation in intervention activities and positive changes in attitudes toward local foods. Increased self-efficacy in food production and preparation. Knowledge: Reported learning about local food, health, and the transfer of information from youth to adults.
Hanson et al., 2011, Federated States of Micronesia [50].	75 households in 2009; 68 households in 2010; 53 households for in-depth interviews; ages ranged from 21 to 70 (mean 53); 50 women and 18 men in 2010.	Residents of Kapinga Village, an urban area on the island of Pohnpei.	Promote local food consumption and PA to improve health and prevent chronic diseases. 1 year. Educational sessions, gardening and cooking activities, and seed and plant distribution. Gardening workshops: how to plant in available containers such as buckets and burlap sacks, proper soil composition, and how to care for and grow seedlings. Participants were provided with cucumber, eggplant, Chinese cabbage, bell pepper seeds, and papaya plants. Technical advice was provided over three months for plant growth. Two-day charcoal workshops covering the advantages of charcoal ovens, such as healthier cooking, cost savings, and environmental benefits, such as lower carbon emissions. 3) Week-long cooking classes: ten recipes focusing on local foods.	Local starch foods: breadfruit, green banana, giant swamp taro, taro, cassava, yam. Local vegetables: banana flower, chaya, puke, spinach, chili leaf, kangkong, basil, beans, eggplant, cabbage, bell pepper. Local fruits (native): banana, mango, apple, watermelon, citrus (calamansi, tangerine, pineapple), guava, papaya, pandanus.	Quantitative data (pre–post intervention): dietary assessment and consumption frequency—seven-day FFQ. Qualitative data (post-intervention): awareness and attitudes toward the intervention and knowledge and beliefs about health and workshops contents—questionnaire and observations.	Dietary intake: Local fruits ↑ from 1.2 to 2.9 mean days/week (*p* < 0.001); local vegetables ↑ from 2.8 to 4.6 mean days/week (*p*< 0.001); 81% of households ate breadfruit at least once a week; 68% ate banana, 57% ate giant swamp taro, 57% ate chaya (a green leafy vegetable), 98% ate mango. Imported vegetables ↑ from 0.7 to 2.0 mean days/week (*p* < 0.001); local fish and seafood ↑ from 2.5 to 4.4 mean days/week. 62% of households consumed reef fish, and 75% imported (canned) fish. Knowledge: Usefulness: learning how to plant and cook healthier meals using parts of plants previously discarded. Learning the benefits of local foods over imported rice and flour. Others: Saving money eating food from their garden. Barriers: Rats eating the seeds and lack of space to grow the foods.
Roche et al., 2017, Ecuador [51].	Quantitative analysis: mothers of children ages 2–6 years (160:98); qualitative analysis: 54 mothers from IG, 19–45 years (mean 29 ± 7 years), and 16 elders involved in the intervention, ages 60–90 (mean 79 ± 8 years).	Quichua women and their children in highland Tungurahua, Ecuador.	*Corazon en Familia*. Promote two Indigenous greens. 1 year. Cooking activities. 12-day participatory cooking sessions at their places with other mothers to prepare recipes with local foods (adding to other dishes, such as soup and egg omelette) and eat together. One elder to promote Quichua language and culture. Cooking clubs particularly promoted two Indigenous greens—stinging nettle/ Urtica dioica L. and round-leaved dock/Rumex obtusifolius L. into children’s diets. CG: mothers residing in similar communities that had not yet started the intervention.	Stinging nettle (*Urtica dioica*), round-leaved dock (*Rumex obtusifolius*), and many others native foods such as pea flour, yellow carrot, fava beans, corn flour beverage, and quinoa.	Quantitative data (pre–post intervention): dietary diversity scores based on the consumption of 23 promoted traditional foods. Weekly and daily nutrient contributions from the two Indigenous greens (stinging nettle and round-leaved dock)—FFQ. Qualitative data (post-intervention): perceptions of the intervention, cultural practices, and impacts on cultural identity—focus groups and interviews.	Dietary intake: ↑ dietary diversity, ↑ consumption of TF. 74% of the mothers in the IG fed their children nettle at least once per month, compared to 21% in the CG. The likelihood of mothers in the IG feeding their children these leafy greens was almost 10 times greater than that of mothers in the CG (adjusted odds ratio (aOR): 9.5; 95% CI: 4.37, 20.21; *p* < 0.001). Dietary diversity scores were higher in the IG (15.0 ± 4.4) compared to the CG (13.4 ± 4.6). Nutrient intake: Nettle and dock contributed to nutrient daily recommended intakes for children 2–6 years old: 6.7% of iron, 27.5% of folate, 8% of vitamin A, 12.3% of vitamin C, 2% of calcium, 1.9% of zinc, and 25.5% of magnesium. Nettle provided 27% of daily folate requirements and 12% of vitamin C. Perceptions: Pride and sense of community, recognition of TF as nutritious option, improved relationships. Intervention promoted community connection, improved relationships between mothers and peers, between mothers and children, and between children and peers. Elders noted the nutritional benefits of TF compared with modern foods.

↑ increased, ↓ reduced, BMI—Body Mass Index, CAB—Community Advisory Board, CG—Control Group, CHO—Carbohydrate, DM—Diabetes, FFQ—Food Frequency Questionnaire, IG—Intervention Group, FSM—Federated States of Micronesia, FV—Fruit and Vegetable, HDL—High-Density Lipoprotein, LDL—Low-Density Lipoprotein, NA—Not Available, PA—Physical Activity, PTN—Protein, RCT—Randomised Controlled Trial, RDI—Recommended Dietary Intake, TAG—Triglycerides, TD—Traditional Diet, TF—Traditional Food, TFS—Traditional Food System, TFFQ—Traditional Food Frequency Questionnaire, WD—Western Diet.

## Appendix C. Participatory Approach Used by the Studies

**Table A3:** Participatory approach used by the studies (*n* = 19).

Participatory Approach	N	%	References
Clearly stated CBPR, CAB, CAG, co-design, and/or steps of the participatory approach.	12	63.2	Kolahdooz et al. [53], Bersamin et al. [54], Blanchet et al. [57], Cueva et al. [41], Cueva et al. [40], McEachern et al. [43], McEachern et al. [42], Englberger et al. [45], Emm et al. [47], Stroink and Nelson [48], Kaufer et al. [49], and Roche et al. [51].
Mentioned participatory approach in some of the steps without detailed information.	3	15.8	Oppezzo et al. [55], Iwasaki-Goodman [39], and Hanson et al. [50].
No mention of participatory approach.	3	15.8	Lewis et al. [56], Leslie [44], and Mbhatsani et al. [46].
Conducted by community members.	1	5.3	Schmid et al. [52].

CBPR—Community-Based Participatory Research, CAB—Community Advisory Board, CAG—Community Advisory Group.

## Appendix D. Quality Assessment

**Table A4:** Quality assessment based on Mixed Method Appraisal Tool—MMAT (*n* = 19).

Study	Screening Questions (for All Types)		1. Qualitative Studies					2. Quantitative Randomised Controlled Trials					3. Quantitative Non-Randomised Studies					4. Quantitative Descriptive Studies					5. Mixed Methods					Score (%)
	S1	S2	1.1	1.2	1.3	1.4	1.5	2.1	2.2	2.3	2.4	2.5	3.1	3.2	3.3	3.4	3.5	4.1	4.2	4.3	4.4	4.5	5.1	5.2	5.3	5.4	5.5	
Leslie [44]	y	y	na	na	na	na	na	na	na	na	na	na	na	na	na	na	na	c	c	y	c	c	na	na	na	na	na	42.9
Schmid et al. [52]	y	y	na	na	na	na	na	na	na	na	na	na	na	na	na	na	na	y	y	y	c	y	na	na	na	na	na	85.8
Stroink and Nelson [48]	y	y	y	y	y	y	c	na	na	na	na	na	na	na	na	na	na	y	y	y	c	c	y	y	y	y	y	82.3
Englberger et al. [45]	y	y	na	na	na	na	na	na	na	na	na	na	na	na	na	na	na	y	y	c	c	c	na	na	na	na	na	57.1
Kaufer et al. [49]	y	y	y	c	c	c	c	na	na	na	na	na	na	na	na	na	na	y	y	y	y	y	y	c	c	c	c	52.9
Hanson et al. [50]	y	y	y	y	y	y	y	na	na	na	na	na	na	na	na	na	na	c	c	y	c	y	y	y	y	y	y	82.3
Kolahdooz et al. [53]	y	y	na	na	na	na	na	na	na	na	na	na	y	y	y	c	y	na	na	na	na	na	na	na	na	na	na	85.8
Iwasaki-Goodman [39]	y	y	y	y	y	y	y	na	na	na	na	na	na	na	na	na	na	na	na	na	na	na	na	na	na	na	na	100
Roche et al. [51]	y	y	y	y	y	y	y	c	c	c	c	c	na	na	na	na	na	na	na	na	na	na	y	y	y	y	c	64.7
Mbhatsani et al. [46]	y	y	na	na	na	na	na	na	na	na	na	na	na	na	na	na	na	y	c	y	c	c	na	na	na	na	na	57.1
Bersamin et al. [54]	y	y	na	na	na	na	na	na	na	na	na	na	c	y	c	c	c	na	na	na	na	na	na	na	na	na	na	42.9
Emm et al. [47]	y	y	na	na	na	na	na	na	na	na	na	na	na	na	na	na	na	c	c	y	c	y	na	na	na	na	na	57.1
Cueva et al. [41]	y	y	y	y	y	y	y	na	na	na	na	na	na	na	na	na	na	na	na	na	na	na	na	na	na	na	na	100
Cueva et al. [40]	y	y	y	y	y	y	y	na	na	na	na	na	na	na	na	na	na	na	na	na	na	na	na	na	na	na	na	100
McEachern et al. [42]	y	y	y	y	y	y	y	na	na	na	na	na	na	na	na	na	na	na	na	na	na	na	na	na	na	na	na	100
Oppezzo et al. [55]	y	y	na	na	na	na	na	y	y	y	c	y	na	na	na	na	na	na	na	na	na	na	na	na	na	na	na	85.7
Blanchet et al. [57]	y	y	na	na	na	na	na	na	na	na	na	na	na	na	na	na	na	y	y	y	c	y	na	na	na	na	na	85.7
McEachern et al. [43]	y	y	y	y	y	y	y	na	na	na	na	na	na	na	na	na	na	na	na	na	na	na	na	na	na	na	na	100
Lewis et al. [56]	y	y	na	na	na	na	na	y	y	y	no	y	na	na	na	na	na	na	na	na	na	na	na	na	na	na	na	85.7
																											**Average**	**77.3**

y—yes, c—can’t tell, na—not applicable.
